# A Challenging Decision: Identifying and Managing Hepatic Hydrothorax in the Setting of a Pancreatic Neuroendocrine Tumor

**DOI:** 10.1155/crgm/9397326

**Published:** 2025-12-17

**Authors:** Sultan Ahmed, Ayaz Gen, Abu Fahad Abbasi, Saad Rashid, Sanya Siraj, Altaf Dawood

**Affiliations:** ^1^ Department of Internal Medicine, Mercyhealth GME Consortium, Javon Bea Hospital, Rockford, Illinois, USA; ^2^ Department of Gastroenterology, Mercyhealth GME Consortium, Javon Bea Hospital, Rockford, Illinois, USA

**Keywords:** ascites, hepatic hydrothorax, neuroendocrine tumor, pancreatic

## Abstract

Neuroendocrine tumors (NETs) are rare neoplasms composed of neuroendocrine cells and seen in approximately 2% of malignancies in the United States. These are often underdiagnosed due to nonspecific presentations early in the disease course. Although found primarily in the gastrointestinal tract, lungs, and pancreas, these tumors can be seen anywhere in the body. The following case highlights an unusual presentation of a NET and subsequent management of hepatic hydrothorax in the setting of obstruction rather than decompensated liver cirrhosis.

## 1. Introduction

Neuroendocrine tumors (NETs) comprise 0.5% of all newly diagnosed malignancies and 2% of malignancies in the United States. Often found in the gastrointestinal tract, lungs, and pancreas, many of these malignancies arise sporadically. With varying presentations at onset and diagnosis, these tumors are considered functional if they have release of vasoactive substances, such as serotonin. Regarding pancreatic NETs, several functional malignancies have been noted including insulinomas, gastrinomas, pancreatic polypeptide–secreting tumors, vasoactive‐intestinal peptide–secreting tumors, glucagonomas, and somatostatinomas. Each of these malignancies are associated with overlapping and individual clinical presentations [1, 2]. Commonly, however, these tumors are nonfunctional and found incidentally due to nonspecific symptoms of abdominal distension and dyspnea secondary to mass effect from the neoplasm. The following is a case of an unusual presentation of a NET and subsequent hepatic hydrothorax (HH), in the presence of early onset portal hypertension, without evidence of cirrhosis, due to mass effect from the malignancy.

## 2. Case Presentation

A 65‐year‐old female with a past medical history of Gastroesophageal Reflux Disorder (GERD) presented to the emergency department (ED) with a three‐month history of fatigue, dyspnea, bilateral lower extremity swelling up to the thighs, and abdominal distention. Prior to presentation, she had seen outpatient gastroenterology for the evaluation of intermittent, foul‐smelling diarrhea for the past six months with a weight loss of 30 lbs. Workup including lipase, celiac total, and transglutaminase IgA levels is unremarkable. She was managed with pancreatic enzyme replacement therapy for supposed insufficiency without significant improvement. Colonoscopy was recommended but not performed. Given the worsening shortness of breath with unclear etiology, she presented for further evaluation. In the ED, the patient was afebrile and tachycardic. Exam was notable for cachexia, crackles at the lung bases, abdominal distension, and bilateral lower extremity grade 2 pitting edema. Pertinent negatives include anicteric sclera, no hepatomegaly, spider angiomas, or telangiectasias.

Initial labs were notable for glucose of 141, blood urea nitrogen of 14, creatinine of 0.5, sodium of 133, chloride of 95, potassium of 2.7, magnesium of 0.9, total bilirubin of 0.4, aspartate transaminase of 19 and alanine transaminase of 26, albumin of 3.2, white blood cell count of 6.4, hemoglobin of 13.9, mean corpuscular volume of 89, and platelet count of 471. Lactate dehydrogenase (LDH) level was normal. Initial imaging studies included computed tomography (CT) of the chest, abdomen, and pelvis. Imaging was significant for massive right pleural effusion, with compression upon the right and left atrium secondary to mass effect, subtotal collapse of the right lung with left mediastinal shift, and a medial, left lower lobe subsegmental pulmonary embolism (PE). Abdominal imaging showcased four‐quadrant ascites (Figure 1(a)), heterogenous mass‐like thickening of the pancreatic head and uncinate process concerning for pancreatic neoplasm encasing the superior mesenteric artery, compression of the superior mesenteric vein (SMV) (Figure 1(b)), and considerable retroperitoneal lymphadenopathy. Gastroenterology and general surgery were consulted.

Figure 1CT abdomen/pelvis image demonstrating four‐quadrant ascites (a). CT abdomen/pelvis image displaying thickening of pancreatic head and uncinate process, concerning for neoplastic growth (b).

**Figure:**
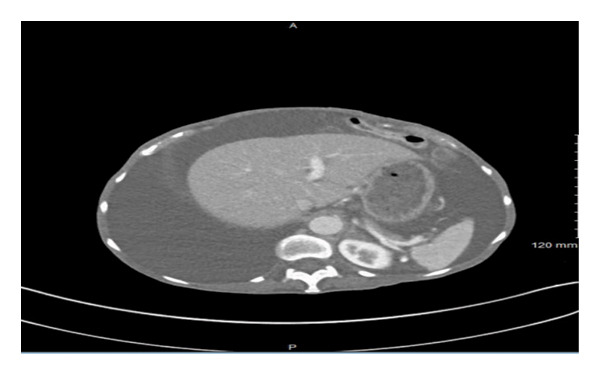
(a)

**Figure:**
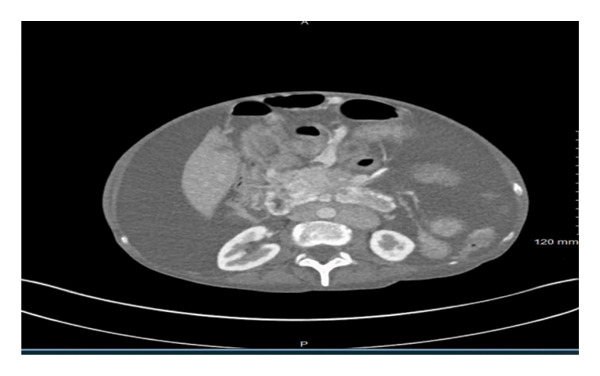
(b)

Thoracostomy was performed with placement of a chest tube in the ED, and the patient was started on heparin in the setting of a PE. Fluid studies showed an LDH of 59 U/L, protein of 1.5 g/dL, cell count of 157, and triglycerides of 343. Pleural protein:serum protein ratio was 0.3, and pleural LDH:serum LDH ratio was 0.24, consistent with a transudative effusion. CA 19‐9 and hepatitis panel were negative. The patient was maintained on a clear liquid diet early on. Due to poor oral intake, malnourishment, and BMI of 12, total parenteral nutrition was required.

The patient underwent abdominal paracentesis with interventional radiology (IR). Fluid studies showed a serum albumin‐ascites gradient (SAAG) of 2.3 suggestive of portal hypertension. Lymph node biopsy was performed by IR and, therefore, endoscopy was not pursued. During the procedure, an accordion drain was placed to reduce intra‐abdominal pressure to allow for a SMV stent to be placed. Stenting was later performed to reduce pancreatic mass effect and relieve portal hypertension. Biopsy showed NET staining positive for chromogranin A (CgA). Oncology was consulted and recommended care at a tertiary facility that specialized in NETs. Patient was given long‐acting octreotide. anticoagulation with apixaban 5 mg twice daily was decided upon prior to discharge given the patients PE, and other medications included addition of simethicone and spironolactone. Pathology indicated metastatic grade 2, well differentiated NET, likely of pancreatic origin, and Ki67 of 4.1% (Figure 2). The tumor was considered functional due to vasomotor symptoms and diarrhea. Per oncology recommendation, patient was started on oral chemotherapy with capecitabine and temozolamidem with continuation of long‐acting octreotide. Postdischarge, the patient had a positron emission tomography scan which redemonstrated the pancreatic neoplasm with findings of hepatic metastases, paraoesophageal nodal metastases, and supraclavicular, mesenteric, and retroperitoneal somatostatin receptor positive metastatic lymphadenopathy. The patient met with surgical oncology but was deemed to be a poor candidate for surgery and it was decided to continue chemotherapy and octreotide. After three cycles of chemotherapy, spanning approximately four months, repeat imaging failed to show significant nodal response. It was decided to maintain monthly octreotide for symptom management and quality of life measures. The patient was recommended to have continued follow up with repeat imaging in three months to assess disease status and further guide management.

Figure 2Histopathology of the lymph node biopsy suggestive of a NET. Stains provided include H&E (a), synaptophysin (b), and Ki‐67 (c).

**Figure:**
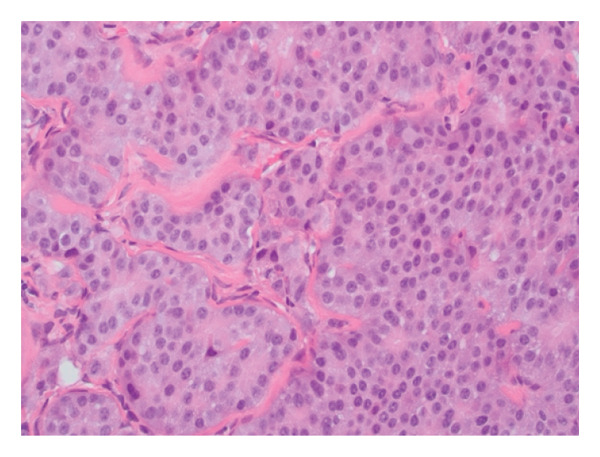
(a)

**Figure:**
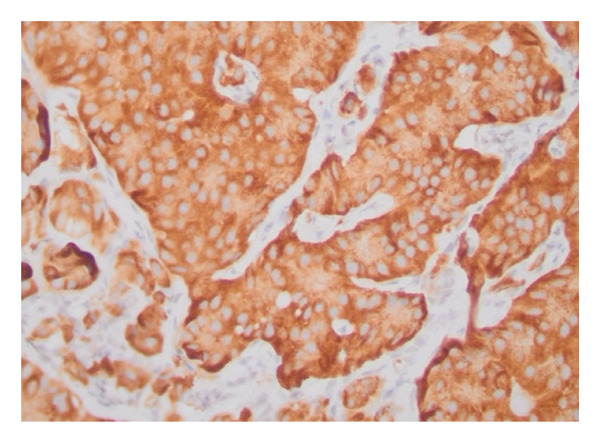
(b)

**Figure:**
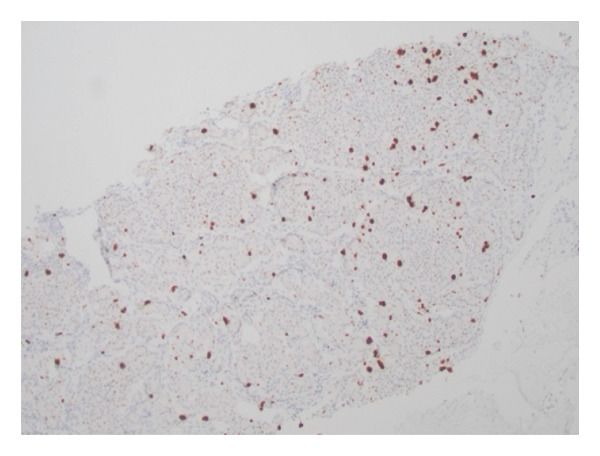
(c)

## 3. Discussion

NETs commonly have a delayed diagnosis due to nonspecific presenting symptoms such as flushing, abdominal pain, or diarrhea. In a prospective study by Ter‐Minassian et al., the median timeline of symptom duration prior to presentation is 3.4 months, and common initial diagnoses included irritable bowel syndrome, inflammatory bowel disease, and gastritis [3, 4]. Most NETs are considered nonfunctional, however, with only 10% of documented neoplasms resulting in carcinoid syndrome. Presentation and initiation of diagnostic schema for patients is likely in response to the sequelae of mass effect from the tumor or metastatic disease [5]. This patient’s case was unique because not only was the tumor functional, as demonstrated by the vasomotor symptoms and diarrhea, but she also presented with mass effect secondary to the pancreatic NET compressing the SMV, leading to duodenal varices and abdominal ascites with HH.

Given the patient’s initial complaint of dyspnea, a myriad of differentials warranted an investigation. The differential for abdominal ascites includes heart failure, nephrotic syndrome, cirrhosis with portal hypertension, cancer, and severe pancreatitis. The absence of tobacco use, documented pulmonary disease, evidence of arrhythmia, coronary artery disease, or heart failure narrows the etiologies. When considering alarm symptoms such as weight loss and diarrhea, imaging is a prudent diagnostic step. In her case, CT chest/abdomen/pelvis showed a massive right pleural effusion, medial left lower lobe segmental pulmonary emboli, massive four‐quadrant ascites, and heterogenous mass‐like thickening of the pancreatic head and uncinate process concerning for a pancreatic neoplasm. Right upper quadrant ultrasound had findings of hepatofugal flow in the right portal vein, indicative of early onset portal hypertension.

For diagnostic and therapeutic purposes, a chest tube was placed, and fluid sample testing was suggestive of a transudative effusion. Transudative causes of pleural effusions are congestive heart failure, nephrotic syndrome, and cirrhosis, whilst exudative causes include pneumonia, cancer, or tuberculosis [6]. Abdominal ascites, with hepatofugal flow on ultrasound and a transudative pleural effusion, led to the consideration of HH.

HH is an uncommon cause of transudative pleural effusion, seen in only 2%‐3% of all cases of pleural effusions. The effusion occurs in the setting of decompensated liver cirrhosis, with no active cardiopulmonary pathology [7, 8]. This patient had mass effect with compression of the SMV, obstructing venous outflow from the intestines and causing duodenal varices. The buildup of pressure propagates to other mesenteric vessels that feed the portovenous system and lends to portal hypertension. Pressure in the splanchnic capillaries disrupts fluid balance with elevated hydrostatic pressure, causing leakage of fluid into the abdomen [9]. Translocation of ascitic fluid through defects in the diaphragm is believed to be the origin of the pleural effusion. Similar mass effect has been seen with other cases of gastrointestinal tumors and can often be the cause of presentation for evaluation [10, 11]. Therapy for HH is aimed at fluid and salt restriction combined with diuresis. Use of a chest tube is not indicated and can cause electrolyte imbalance due to persistent drainage of fluid. Other interventions include therapeutic thoracentesis, catheter use, and TIPS procedure [7]. This patient had a chest tube placed on initial presentation prior to diagnosis of HH and continued to have significant output. Initially in the hospital course, low blood pressure prevented consistent use of diuretics. During her stay, the patient underwent placement of an accordion drain, which was intended to reduce ascites, to allow for stenting of the SMV (Figure 3). Due to the mechanism discussed previously, compression of the SMV would presuppose ascitic fluid buildup. Although TIPS can be considered to bypass portal hypertension, in this patient, relief of obstruction resolved the increased portal pressure. Therefore, no further intervention was required. Reported cases of cystic liver disease, and similarly with infiltrative processes, have been associated with increased portal pressure, and the approach to therapy here can include TIPS or liver transplant [12].

Figure 3Compressive causes of ascites were confirmed on IV venogram which showed that the patient had large and extensive duodenal varices (a). Balloon inflation at the SMV partially alleviated the occlusion (b); therefore, stenting of the SMV for both relief of symptoms and management of HH was performed (c).

**Figure:**
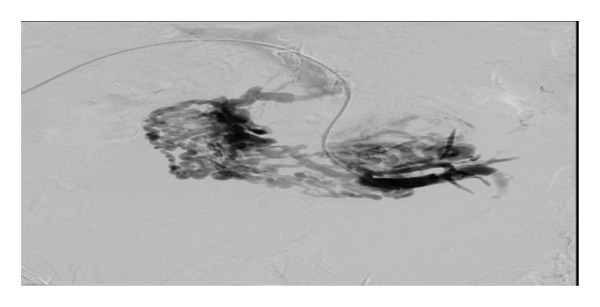
(a)

**Figure:**
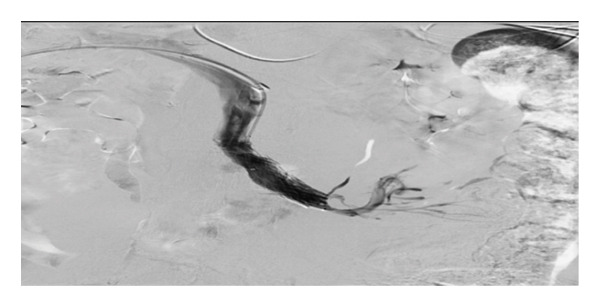
(b)

**Figure:**
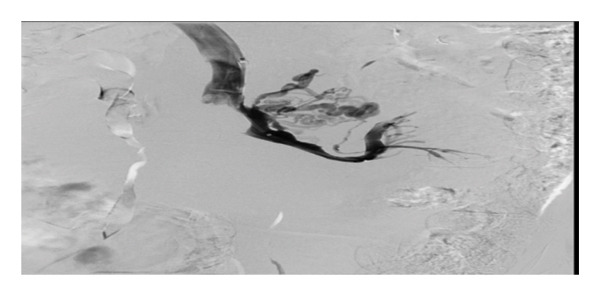
(c)

This patient’s dyspnea was multifactorial and improved with relief of her pleural effusion and ascites. Another aspect of her shortness of breath, however, was her PE. Presence of cancer can result in a hypercoagulable state with a higher risk of venous thromboembolism (VTE) than in the average population and a reported 4–13 times higher rate of incidence in patients with metastatic disease. Furthermore, the incidence of VTE has been associated with an increased risk of one‐year mortality [13, 14]. Guidelines suggest that patients be stratified for risk of VTE to determine if anticoagulation is indicated. Given the patient’s pancreatic mass, she had a score of 2 per the Khorana scale and was considered intermediate risk with a thrombosis rate of 1.8%–2% [15]. Due to the PE on presentation, anticoagulation was already indicated. Anticoagulation is generally recommended for at least three months or until chemotherapy is stopped. For patients with active malignancy, continued anticoagulation is recommended. Although bleeding is a concern, a Cochrane review of nine randomized controlled trials noted, for ambulatory patients receiving chemotherapy, a reduction in risk of VTE without a significant rise in risk of bleeding [16].

Diagnosis of NETs requires a multifaceted approach with tissue pathology testing, biochemical testing, and imaging [17]. Biochemical testing can involve the use of a biomarker known as CgA. Sensitivity and specificity for this test can vary based on the type of malignancy, level of differentiation, and the assay/kit [18]. For NETs, the sensitivity of the test can range from 60% to 80% and specificity can range from 70% to 100%. In a single‐center retrospective study correlating CgA levels with patient prognosis, high baseline CgA levels prior to any treatment, regardless of NET type or functionality, were associated with worse overall survival [17]. An increase of CgA > 40% from baseline in serial follow‐up was associated with a high risk of tumor progression or recurrence [19].

Another aspect of diagnosis is tumor grade. Per the World Health Organization, Ki‐67 is an important prognosticating factor; estimated by microscopic examination of tumor cells and counting the number of Ki‐67 positive cells, an index of < 3% is associated with a Grade 1 (G1) tumor, 3%–20% is Grade 2 (G2), and > 20% is Grade 3 (G3) [20, 21]. Lower scores are associated with favorable prognosis. Furthermore, an extremely high Ki‐67 proliferative index, > 70%, is usually indicative of neuroendocrine carcinomas (NEC). Improved mortality outcomes have been noted for NETs over NEC [22]. In a retrospective study by Pape et al., CgA positivity, with low Ki‐67 (cell proliferation marker), was associated with a favorable prognosis [17, 23].

Management of pancreatic NETs is multidisciplinary, with both systemic and surgical interventions. For patients with G1 and G2 tumors, even in the case of metastatic disease, surgery with intent to cure is an initial consideration [24]. For patients who are somatostatin‐receptor positive with G1 to G2 disease, long‐acting somatostatin analogs are utilized for tumor growth control. If significant tumor burden is present, with hepatic involvement, hepatic arterial embolization can be an adjunct to the somatostatin analog [25]. For G2 disease with a Ki‐67 index of 10%–20% or concern for disease progression, peptide‐receptor radionuclide therapy and a somatostatin analog have improved progression‐free survival [26]. Patients who are not positive for somatostatin receptor uptake can be managed initially with everolimus or cabozantinib [27, 28]. Systemic chemotherapy is recommended in low‐grade tumors with high tumor burden or significant progression. Streptozocin/5‐fluorouracil and temozolomide or combination with capecitabine are regimens indicated for G1, G2, or G3 NETs. Follow‐up of patients with low‐grade tumors can involve measurement of biochemical markers every 3–9 months and dedicated imaging [24].

Prior to discharge, this patient was started on long‐acting octreotide [23]. Outpatient workup with positron emission tomography redemonstrated the pancreatic neoplasm, with findings of multiple hepatic metastases, para esophageal nodal metastases, and several supraclavicular, mesenteric and retroperitoneal somatostatin receptor‐positive metastatic nodes. Due to poor conditioning status and evidence of significant metastatic disease, she was determined to be a poor surgical candidate. Per oncology, she was recommended to continue with octreotide, spironolactone, and a combination of capecitabine and temozolomide therapy. Apixaban was continued due to the high risk for a repeat thromboembolic event. Follow‐up monitoring involved blood counts, chemistry/metabolic panels, thyroid checks, chromogranin, and serotonin levels every 3–5 months. She received three cycles of chemotherapy with no significant lymph node response on repeat imaging. Oncology eventually recommended cessation of chemotherapy. The patient was maintained on octreotide infusions for quality‐of‐life measures, as she reported resolved diarrheal symptoms.

When comparing this patient’s case to the general population, analysis of cases of pancreatic NETs from the cancer registries of the Surveillance, Epidemiology, and End Results (SEER) program noted a median overall survival for all cases to be 28 months. Higher‐grade tumors were associated with worse outcomes versus G1 and G2 [29]. When considering mass effect, if resection of a locally advanced tumor is planned, it can be made difficult as the tumor can have an association with mesenteric vessels and increase the risk of vascular compromise [30]. Although the patient had characteristics of a favorable prognosis, due to poor functional status, she was a poor surgical candidate and did not demonstrate a significant response to chemotherapy. Given the functional status of her tumor with subsequent mass effect, her presentation was unique and required a significant multidisciplinary approach for both initial diagnosis, management, and subsequent follow‐up after stabilization.

In conclusion, NETs are rare malignancies with nonspecific presentations that are commonly misdiagnosed on presentation. This patient presented with HH, in the absence of cirrhosis, due to mass effect from a pancreatic NET causing compression of the SMV. Although cases have been documented that show pleural fluid accumulating in the setting of pulmonary NETs, few reports showcase the correlation of pancreatic NETs with the presentation of HH to our knowledge. We hope this patient’s case can contribute to the literature that serves to advance understanding of this rare and unique class of neoplastic disease.

## Consent

Written informed consent was obtained from the patient for the publication of this case report.

## Conflicts of Interest

The authors declare no conflicts of interest.

## Funding

No funding was received for this manuscript.

## Data Availability

Data sharing is not applicable to this article as no new data were created or analyzed in this study.
